# Ultra-wideband manipulation of electromagnetic waves by bilayer scattering engineered gradient metasurface

**DOI:** 10.1039/c7ra11953d

**Published:** 2018-04-09

**Authors:** Yinghui Guo, Jing Yan, Mingbo Pu, Xiong Li, Xiaoliang Ma, Zeyu Zhao, Xiangang Luo

**Affiliations:** State Key Laboratory of Optical Technologies on Nano-Fabrication and Micro-Engineering, Institute of Optics and Electronics, Chinese Academy of Science P. O. Box 350 Chengdu 610209 China lxg@ioe.ac.cn; University of Chinese Academy of Sciences Beijing 100049 China

## Abstract

Realizing effective scattering manipulation in broad operation band is a long pursuit. Here, we present an ultra-broadband metasurface to manipulate the scattering of electromagnetic waves based on spin–orbit interaction induced virtual shaping. The spin conversion efficiency is over 90% from 4.9 GHz to 22.8 GHz accompanied by a abrupt phase shift covering 0–2π, which is dependent on the orientation of the subwavelength of building blocks. Both Bessel- and triangular-type virtual profile shapes are developed for electromagnetic illusion and camouflage. Simulation and experimental results demonstrate that the significant backward RCS reduction of 10 dB is obtained from 5.5 to 22.5 GHz. The significant improvement in working bandwidth can be attributed to: the application of the dispersionless phase–shift properties of P–B phase, two-dimensional dispersion management methodology and catenary shaped local field enhancement in the metallic gap. The proposed design may have the potential applications in eletromagnetic manipulation and object detection.

## Abstract 2

Ultra-broadband electromagnetic illusion is realized by spin–orbit interaction of two-dimensional dispersion engineered metasurfaces with catenary-shaped local field enhancement.

## Graphical abstract

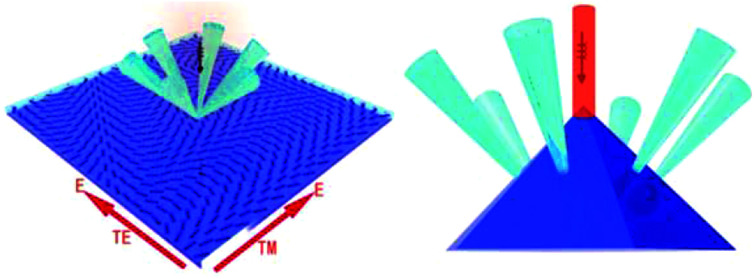

## 1. Introduction

Recently, metamaterials composed of subwavelength resonant structures have attracted considerable attention, with representative applications including color filtering,^1^ super-resolution imaging,^2,3^ and lithography.^4^ With the emergence of metamaterials, strenuous research efforts have been made to manipulate the scattering of electromagnetic waves. Transformation optics using gradient-index media is a typical means to make the object inside the region invisible.^5^ However, the material parameters of cloak required are rigorous, significantly restricting their practical applications. A quasi-conformal mapping method^6^ was subsequently proposed for simplifying the design and minimizing absorption losses. Nevertheless, metamaterial-based mechanisms still remain volumetric and are hard to scale up.

Compared with three-dimensional (3D) metamaterials, two-dimensional (2D) metasurface relax the strict fabrication requirement and thus has promoted the rapid development of ultrathin flat optical devices since the metasurface assisted refraction and reflection law (MLRR) is established.^7,8^ These landmark achievements could open a door for the optical engineering at the subwavelength scale, *i.e.*, for Engineering Optics 2.0,^9^ and provide alternatives to traditional approaches based on bulky optical components.^10–18^ For example, distinct from the electromagnetic absorption in subwavelength structures,^19,20^ scattering engineering based on metasurface offer an alternative method for low delectability,^21–24^ which could be considered as a virtual shaping technology.^25^ However, most existing metasurfaces suffer from a limited operation bandwidth.

Enormous research efforts have been proposed to broaden the working bandwidth of metasurfaces.^26–31^ One typical method is cascading multiple metasurfaces at a cost of increased device thickness and weight. A more effective method to broaden working bandwidth is dispersion management which can approach the bandwidth-thickness limits.^32–36^ In ref. 32, one-dimensional dispersion management was used to achieve polarization conversion with the bandwidth ratio over 3 : 1. Subsequently, bandwidth ratio over 5 : 1 was achieved by 2D dispersion management.^33,34^ Recently, a broadband metasurface for simultaneous thermal infrared invisibility and holographic illusion has been demonstrated.^37^

In this paper, a meta-mirror with bilayer metasurfaces is adopted to realize ultra-wideband scattering manipulation of EM waves. Based on the principle of Pancharatnam–Berry (P–B) phase, dispersionless 0–2π phase discontinuity could be realized by adjusting the orientation of the building blocks. Following the MLRR,^7^ various virtual shapes with low backward radar cross section (RCS) are developed. By virtue of 2D dispersion management of bilayer metasurfaces, ultra-wideband backward RCS reduction are demonstrated through numerical simulations and experiments in microwave regime. The mechanism of the meta-mirror to broaden the working bandwidth mainly depends on the 2D dispersion engineering and the dispersionless geometric phase. The present structure provides a very effective method for the achievement of ultra-broadband electromagnetic manipulation.

## 2. Unit cell design and bandwidth optimization

MLRR provides a new idea for controlling electromagnetic waves,^10,11^ where the degree of freedom to control the waves is enhanced by introducing the phase discontinuity over the scale of the wavelength. A smart method to introduce the achromatic phase discontinuity refers to the space-variant Pancharatnam–Berry (P–B) phase based on the photonic spin–orbit interaction (PSOI).^38,39^ As a novel PSOI platform, optical catenary has been proposed for perfect orbit angular quantum (OAM) generation^38^ and 3D colorful hologram.^39^ When a circularly polarized light illuminates on a reflective half-wave plate, the reflected cross-polarized lights will pick a phase retardation, which can be written as:^38^

1 *Φ*(*x*, *y*)=2*σα*(*x*, *y*)

where, *σ* = ±1 denote the helicity of the incident light and *α*(*x*, *y*) is orientation of the subwavelength building blocks. Since the P–B phase is accompanied by spin conversion, the operation bandwidth of metasurface is ultimately limited by the polarization conversion efficiency, and thus an ultra-broadband polarization convertor is highly desired.

**Fig. 1:**
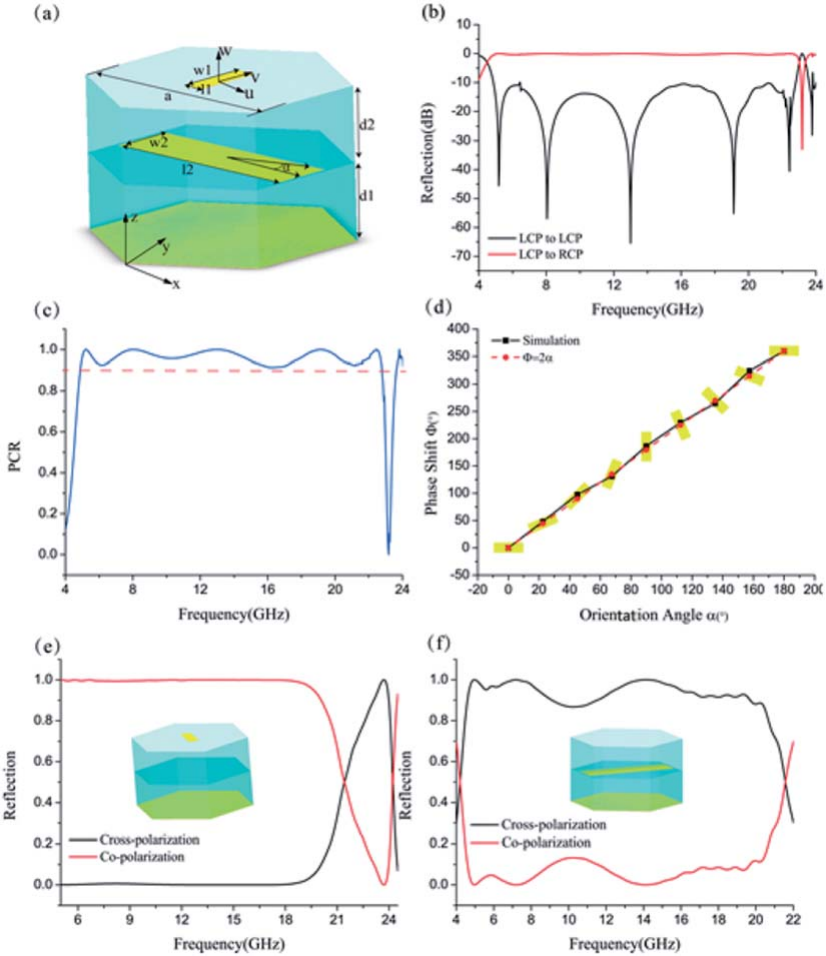
Schematic of the bilayer metasurfaces based on spin–orbit interaction. (a) The schematic of proposed ultra-broadband polarization converters. (b) Simulated reflectance for cross-polarization and co-polarization under normal circularly polarized illumination. (c) Polarization conversion ratio (PCR) of the unit cell. (d) Phase shift as a function of orientation angle at 13 GHz. (e) and (f) Illustrate the simulated circular polarization conversion performance with single upper metasurface and single lower metasurface configuration.

As shown in Fig. 1(a), we proposed a meta-mirror composed of bilayer metasurfaces separated from a metallic reflection plane. Each metasurface is constructed by metallic cut-wire array in a hexagonal lattice with C6 symmetry to enhance the polarization adaptability. The orientations of the cut-wires in each unit are orthogonal to each other to ensure we can make the dispersion engineering in both dimensions.^33,34^ Besides, to broaden the working bandwidth of the metasurface, the substrate thickness *d* is selected as 3 mm (one quarter of the wavelength at 17 GHz) to form a Fabry–Pérot-like cavity with low quality factor so that we can further extend the operation bandwidth.^33^

The polarization conversion performances of meta-mirror was simulated using CST Microwave Studio with unit cell boundary conditions. The dimensions of the unit cell are optimized as *a* = 9 mm, *l*_1_ = 0.9 mm, *w*_1_ = 2.8 mm, *l*_2_ = 8.5 mm, *w*_2_ = 2.4 mm. The metal is taken as PEC and the permittivity of the dielectric spacer is 2.65. According to Fig. 1(b), the reflectance of the co-polarized wave is below −10 dB in an ultra-wide frequency band. The polarization conversion ratio (PCR) is over 90% from 4.9 GHz to 22.8 GHz, *i.e.*, the bandwidth ratio is more than 4 : 1. Besides, when the orientation of subwavelength building varies from 0° to 180°, we can obtain a linear phase shift from 0° to 360°, which is consistent with the principle of P–B phase, as indicated in Fig. 1(d). In order to check the role of each metasurface played in the formation of the ultra-broad operation bandwidth, single MIM metasurface configurations have also been simulated and the results are shown in Fig. 1(e) and (f) in the revised version, from which we can see that the upper and lower metasurface respectively exhibits high cross polarization conversion efficiency in the higher frequency band (21–24 GHz) and lower frequency band (5–21 GHz) due to the geometry differences between them. The whole metasurface can be taken as a hybrid configuration of them.

**Fig. 2:**
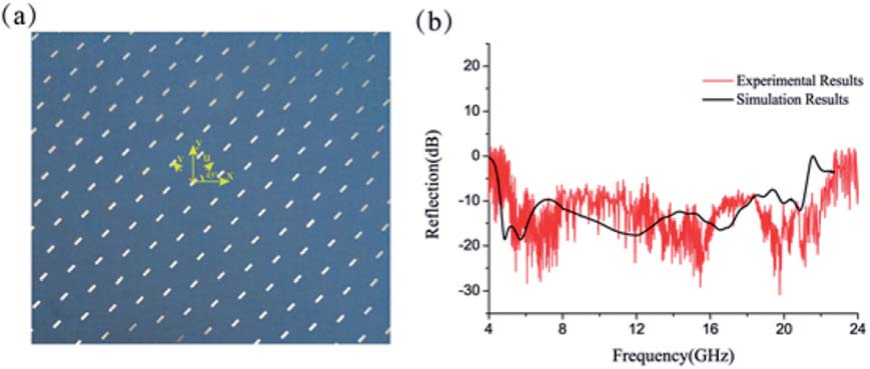
(a) The top view of the sample. (b) Measured and simulated reflection coefficient of co-polarized component under *x*-polarized incidence.

To verify the ultra-wideband polarization conversion characteristics of our structure, a sample with dimension 300 × 300 mm^2^ was fabricated with print circuit board (PCB) technique as shown in Fig. 2(a). The circular polarization conversion (LCP-to-RCP) was replaced by the linear polarization conversion (*x*-to-*y*) in the experiment. We ensure the *y* axis of sample has an angle of 45° with respect to the *x* axis so that the *x*-polarized wave is transformed to *y*-polarization. As depicted in Fig. 2(b), the measured reflection coefficient of the S11 is below −10 dB in the frequency range between 5 GHz and 22.4 GHz.

**Fig. 3:**
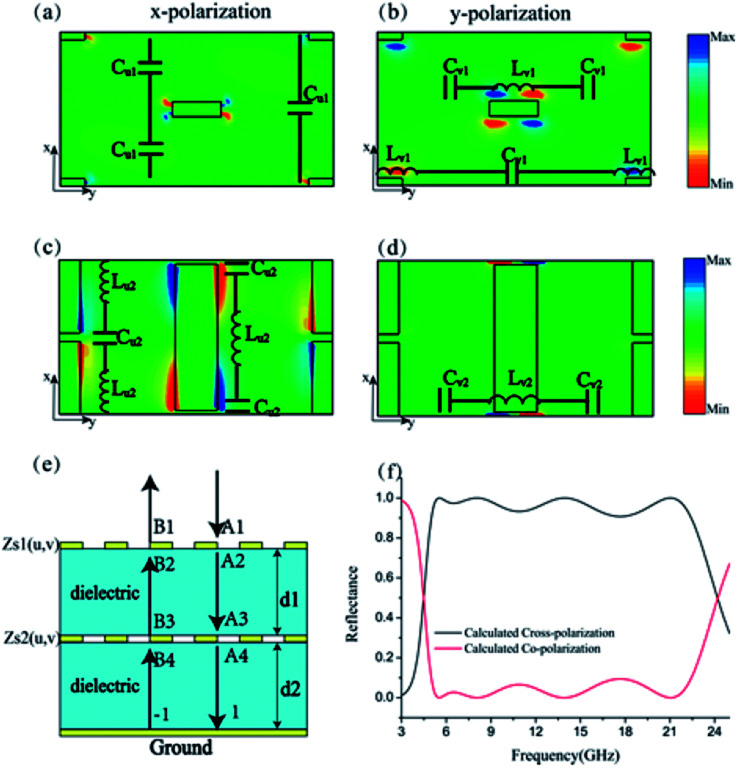
(a) and (c) Illustrate the electric field distribution of the different layers under normal *x*-polarized light illumination at 14 GHz. (b) and (d) Illustrate the electric field distribution of the different layers under normal *y*-polarized light illumination. (e) Schematic of the transmission line model for the bilayer metasurfaces. (f) The calculated results based on transfer matrix method.

Subsequently, equivalent circuit method is utilized to explain the principle of 2D dispersion engineering.^33^ According to the electric field distribution in Fig. 3, we can take the metallic metasurface as a LC circuit, where the inductance *L* origin from the metallic wires while the capacitance *C* stems from the metallic gap. Consequently, the anisotropic impedances of the metasurfaces can be expressed as:2
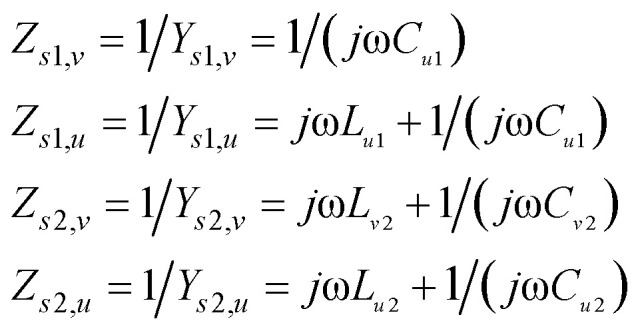
where *u* and *v* represent the orthogonal main axes of the adopted bilayer metasurface, 1 and 2 denote the layer numbers. We can retrieve the circuit parameter by fitting the simulated reflected phases with the calculated results, *i.e.*, (*L*_s1,u_,*C*_s1,v_, *C*_s1,u_) = (5 × 10^−11^ H, 6 × 10^−16^ F, 1.5 × 10^−14^ F) and (*L*_s2,v_, *L*_s2,u_, *C*_s2,v_, *C*_s2,u_) = (3.2 × 10^−9^ H, 2 × 10^−11^ H, 4 × 10^−16^ F, 2 × 10^−13^ F). By utilizing the transfer matrix method (TMM) shown in Fig. 3(e), we can calculate the reflectance of the proposed structure, which is displayed in Fig. 3(f) and consistent with the simulation results in Fig. 1.

**Fig. 4:**
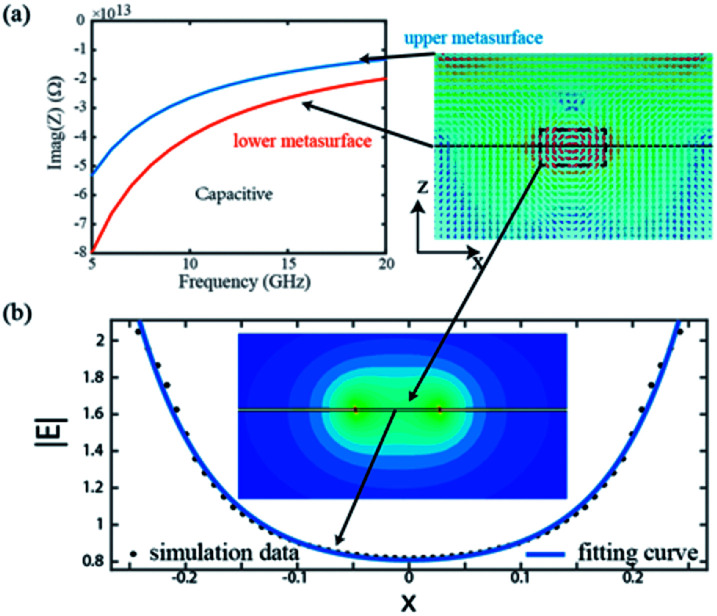
(a) Calculated impedances of the metasurfaces as a function of frequency and corresponding electric field distribution at 12 GHz, which show strong local field enhancement in the metallic gap. (b) The magnitude of electric field distribution in the metallic gap and the evolution curve is fitted by a catenary function.

According to eqn (2), we can deduce the impedances of the impedances and the results are shown in Fig. 4(a), which illustrates the impedances of the metasurfaces are capacitive in the whole operation band. Specially, there are obvious local field enhancement in the metallic gap, as show in the inset of Fig. 4(b). We retrieve the magnitude of the electric field along the metallic gap and show the data in the inset of Fig. 4(c) (black dot). By fitting the these data, we find the local electric field enhancement in the metallic gap follows a catenary function:

3 |*E*| = *a* exp(*bx*) + *c* exp(−*dx*) + *e*

where, *x* is the coordinate position and the fitting coefficients respectively are *a* = *c* = 359.8, *b* = *d* = 15.08, and *e* = 7367 with *R*-square equal 0.9944. Note that the catenary field distribution follow a different function with the catenary of equal phase gradient proposed in ref. 38 and 39.

## 3. Simulation and experimental validation of virtual shaping

**Fig. 5:**
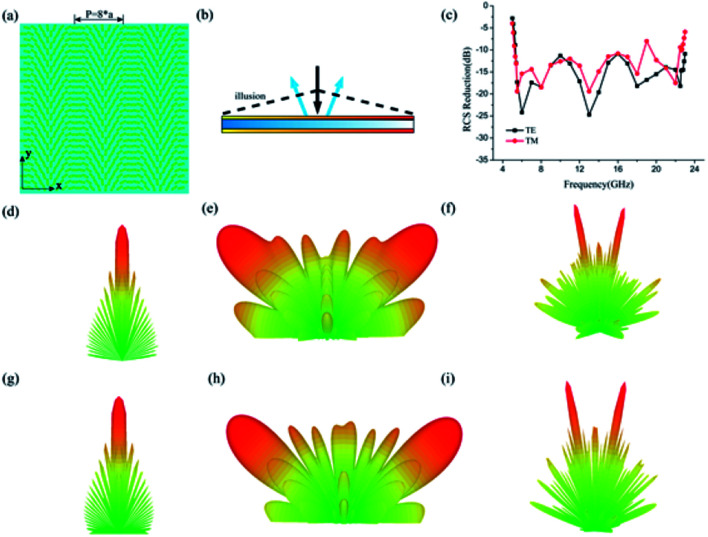
Sketch map of electromagnetic illusion induced by linear varied phase distribution. (a) Schematic of the full-model (b) the virtual shaping induced by anomalous reflect. (c) The simulated results of backward RCS reduction. (d) and (g) Illustrate the 3D scattering patterns of the unpatterned metallic plate under normal incidence under *x*- and *y*- polarized incidence respectively. (e) and (f) Illustrate the 3D scattering patterns of the metasurface under *x*-polarized normal incidence at 5.5 GHz and 22 GHz. (h) and (i) Illustrate the 3D scattering patterns of the metasurface under *y*-polarized normal incidence at 5.5 GHz and 22 GHz.

The key of electromagnetic waves shaping is to engineer the scattering of the waves at will, namely, the proper phase distribution along the interface. After the optimization of the basic element's dimensions, we would switch to design the phase distributions, aiming to direct the reflected wave at an abnormal angle under normal illumination. Initially, a Bessel type phase profile is designed as *Φ*(*x*) = *λk*_0_*x*/*P*, where *P* is a period of the array, *x* is the position on the *x* axis of each basic element and *k*_0_ = 2π/*λ* is the free space wave vector. In accordance with the generalized Snell's law, the reflective beam would be propagation with anomalous reflective angle *θ*_r_ = arc sin(*λ*/*P*). Full-model with dimensions of 280 mm × 280 mm (Fig. 5(a)) was numerically simulated by CST with normal linear polarization. At the same time, an unpatterned metallic plate with the same dimension is also simulated for making a comparison. The corresponding simulated three dimensional (3D) scattering pattern as shown in Fig. 5(d) and (g). Fig. 5(e) and (f) illustrate the simulated 3D far-field scattering patterns of the designed meta-mirror for normal *x*-polarized electromagnetic incident waves at 5.5 GHz and 22.5 GHz, respectively, from which we can clearly observe that the reflective beam is deflected to two symmetrical directions and the virtual shape could be considered as a pyramid. Similar 3D far-field scattering patterns results can also be observed for the *y*-polarized incident, as shown in Fig. 5(h) and (i). The broadband backward RCS reduction features of the virtual shaping caused by the bilayer metasurfaces are shown in Fig. 5(c), proving the powerful abilities in engineering the scattering of electromagnetic waves.

A linear polarized light can be decomposed to two individual circular polarization components with opposite helicity, which can help us better understand the polarization-insensitive performance of our structure. The reflection electric field can be written as:4
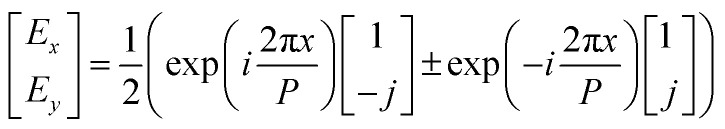
where *x* is the position on the *x* axis of each basic element, the sign ± stand for the *x*-polarization and *y*-polarization respectively. The first and second items in the right of the equation can be considered as the left and right circularly lights.

**Fig. 6:**
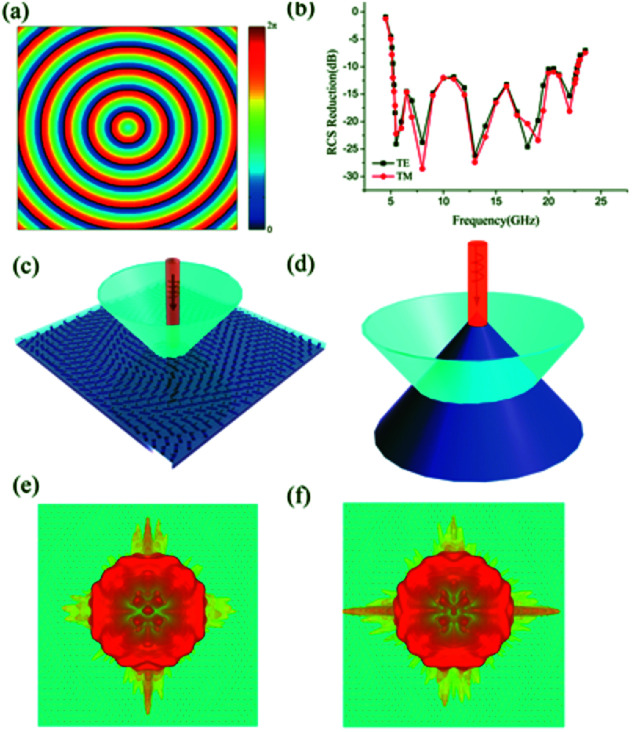
Sketch map of electromagnetic illusion induced by Bessel-type phase distribution. (a) The phase profile of the Bessel-type phase distribution. (b) The simulated results of backward RCS reduction. (c) The scattered beams from the bilayer metasurfaces. (d) The scattered waves from a cone under normal incidence. (e) Illustrate the 3D scattering patterns of structure under normal TE incident waves at 14 GHz. (f) Illustrate the 3D scattering patterns of structure under normal TM incident waves at 14 GHz.

Based on the great freedom of P–B phase, we can design arbitrary phase profiles *Φ* along the interface by rotating the optical axis *α* to achieve the virtual shaping.^25^ According to above design procedure, we arrange a linear planar phase distribution *Φ*(*x*, *y*) = *λk*_0_*r*/*P* on an area of 280 × 280 mm^2^, where 
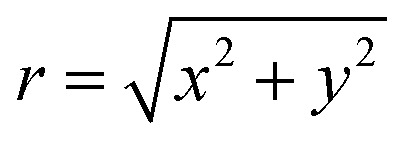
 is the distance between the center of sample and each basic element and *k*_0_ is the wavevector along it. That is the concentric rings each with discrete phase levels *Φ*(*x*, *y*) between 0 and 2π by rotating the orientations of each basic element between 0 and π with the phase profile being presented in Fig. 6(a). Our aim is to ensure that the reflected beam would spread like the ring with different radius, namely, the virtual shape is a cone. Under the normal incidences of linear polarized wave, the simulated 3D scattering patterns of the bilayer metasurface at 14 GHz are shown in Fig. 6(c) and (d). Moreover, based on Fig. 6(b), the backward RCS in 5.2–22.8 GHz is reduced larger than 10 dB compared with a bare metallic plate for both TE and TM and the maximum of the backward RCS reduction exceeds 25 dB around 12 GHz.

**Fig. 7:**
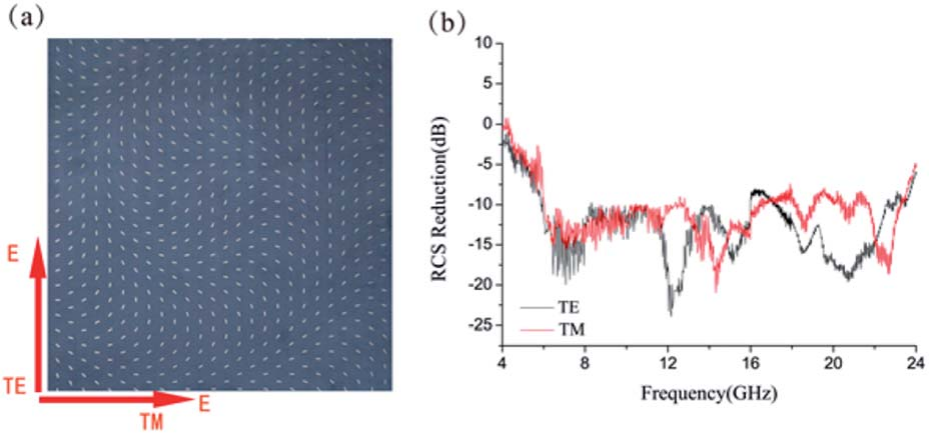
The measurement of the backward RCS reduction. (a) The top view of the sample. (b) Measured results for both TE and TM polarizations.

To verify the ultra-wideband RCS reduction characteristics of the structure, a sample with 300 × 300 mm^2^ was fabricated with print circuit board (PCB) technique, and measured in microwave anechoic chamber. Two standard linearly polarized horn antennas were used for transmitting and receiving the EM waves, and we can change the orientation of linearly polarized horn antennas to measure both the TE and TM polarizations. The RCS measured results of the fabricated sample and metallic flat plates with same dimensions are presented in Fig. 7(b), which display a reasonable agreement, with the consideration of the unavoidable error in the fabricating and measuring process.

**Fig. 8:**
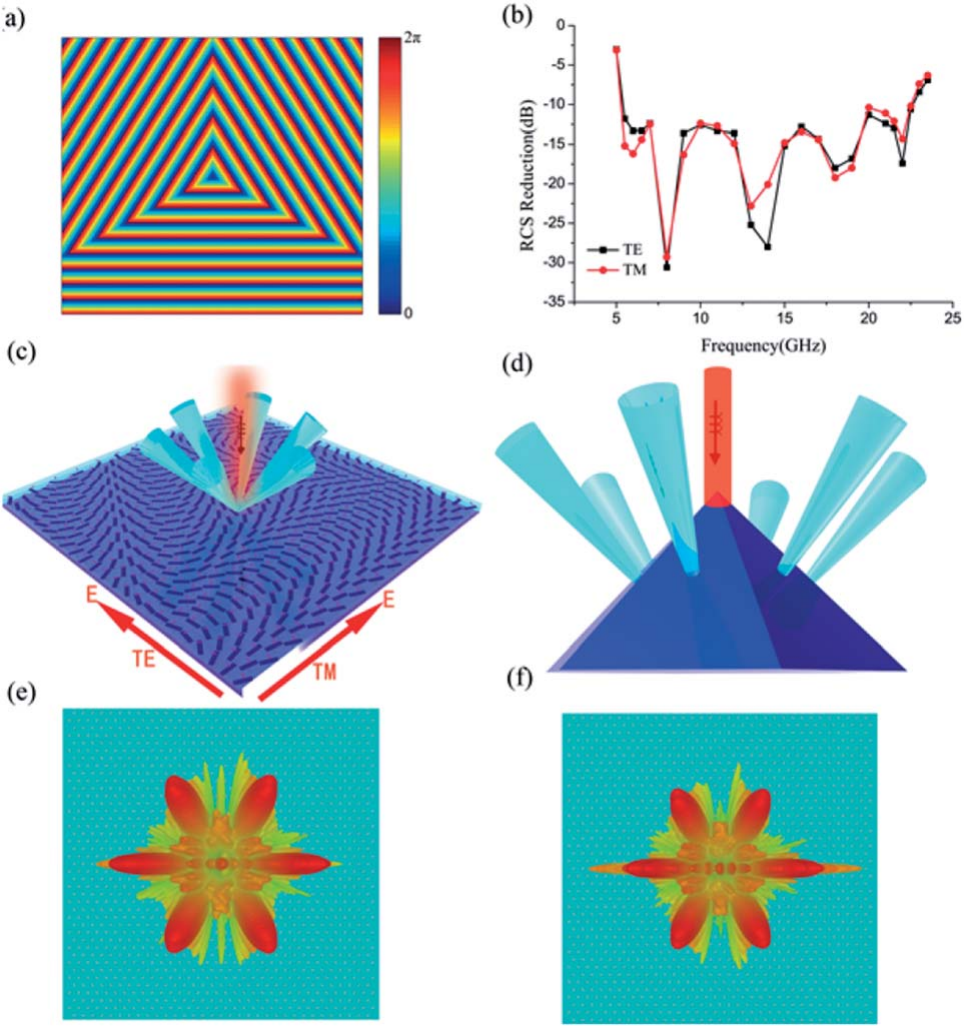
Sketch map of electromagnetic illusion induced by triangle phase distribution. (a) The phase profile of the triangle distribution. (b) The simulated results of backward RCS reduction. (c) The scattered beams from the bilayer metasurfaces. (d) The scattered waves from a hexagonal pyramid under normal incidence. (e) Illustrate the 3D scattering patterns of structure under normal TE incident waves at 14 GHz. (f) Illustrate the 3D scattering patterns of structure under normal TM incident waves at 14 GHz.

To demonstrate the flexibility of our structure in manipulating electromagnetic waves, we designed another phase distributions to achieve the virtual shaping with different detected shape. A planar phase distribution 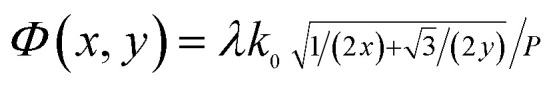 was arranged on the interface to realize the beam diverge into six directions, so the virtual shape become a hexagonal pyramid. The phase profile was designed to achieve the triangle with same dimensions having the same phase distributions, as shown in Fig. 8(a). We checked the performance of our design based on a full-model with dimensions of 280 mm × 280 mm and the 3D far-field scattering results as shown in Fig. 8(e) and (f). Meanwhile, the backward RCS reduction would be larger than 10 dB ranging from 5.5 GHz to 22.5 GHz and the maximum of the backward RCS reduction could reach −30 dB around 8 GHz, as shown in Fig. 8(b). We can clearly observe that the reflection is greatly reduced at the presence of metasurface. Due to the great freedom of P–B phase, the scattered wave power can be redirected to the directions at will.

According to the above simulation results, we demonstrate the arbitrary manipulation capability of the bilayer scattering engineered gradient metasurface in an ultra-broad frequency band. By comparing three backward RCS reduction results, we can observe that the Bessel-type phase distribution and triangle-type phase distribution have better polarization adaptability due to the phase discontinuities along more than one direction. And the Bessel-type phase distribution have better backward RCS reduction performance due to the energy scatter into all direction. From the above analysis, we can understand that the phase discontinuities with more direction can have better performance, it is instructive for our future design work.

## 4. Conclusions

To conclude, we have utilized the bilayer metasurfaces and adopted dispersion engineered photonic spin–orbit interaction to achieve ultra-broadband scattering engineer in the microwave regime. To realize efficient broadband phase modulation, the dispersion management strategy was adopted to ensure ultra-broadband polarization conversion. Three different phase distributions were simulated to show different virtual shapes in a wide band range. The backward RCS of the objects would decrease over 10 dB from 5.5 GHz to 22.5 GHz, eventually reducing the possibility of the target to be detected. Note that, by simultaneously cascading more metasurfaces layers and utilizing the dispersion engineering, larger bandwidth can be obtained. This design provides a strategy for achieving ultra-broadband scattering engineer by adopting dispersion management method and cascading metasurfaces. Moreover, the design presented here can still be improved by utilizing the flexible substrate to achieve the conformal metasurface.

## Conflicts of interest

There are no conflicts to declare.

## Supplementary Material
