# Correction: Ultra-wideband manipulation of electromagnetic waves by bilayer scattering engineered gradient metasurface

**DOI:** 10.1039/c8ra90031k

**Published:** 2018-04-23

**Authors:** Yinghui Guo, Jing Yan, Mingbo Pu, Xiong Li, Xiaoliang Ma, Zeyu Zhao, Xiangang Luo

**Affiliations:** State Key Laboratory of Optical Technologies on Nano-Fabrication and Micro-Engineering, Institute of Optics and Electronics, Chinese Academy of Science P. O. Box 350 Chengdu 610209 China lxg@ioe.ac.cn; University of Chinese Academy of Sciences Beijing 100049 China

## Abstract

Correction for ‘Ultra-wideband manipulation of electromagnetic waves by bilayer scattering engineered gradient metasurface’ by Yinghui Guo *et al.*, *RSC Adv.*, 2018, **8**, 13061–13066.

The journal citations in ref. 10 and 11 were incorrect in the published article. The corrected references are shown below as ref. 1 and 2, respectively.

The Royal Society of Chemistry apologises for these errors and any consequent inconvenience to authors and readers.

## Supplementary Material
